# Supplementary material for: GolpHCat (TMEM87A), a unique voltage-dependent cation channel in Golgi apparatus, contributes to Golgi-pH maintenance and hippocampus-dependent memory
Source: Nat Commun. 2024 Jul 11;15:5830. doi: 10.1038/s41467-024-49297-8 (PMC11239671; doi:10.1038/s41467-024-49297-8)
Supplement: Supplementary file 1 — Supplementary Information [file 41467_2024_49297_MOESM1_ESM.pdf]

Supplementary Information

GolpHCat (TMEM87A), a unique voltage-dependent cation channel in Golgi apparatus, contributes to Golgi-pH maintenance and hippocampus-dependent memory

Hyunji Kang,[1,2,7] Ah-reum Han,[3,7] Aihua Zhang,[4,7] Heejin Jeong,[5] Wuhyun Koh,[1] Jung Moo Lee,[1] Hayeon Lee,[1] Hee Young Jo,[5] Miguel A. Maria-Solano,[4] Mridula Bhalla,[1] Jea Kwon,[1] Woo Suk Roh,[1] Jimin Yang,[3] Hyun Joo An,[5] Sun Choi,[4,*] Ho Min Kim,[3,6,*] C. Justin Lee[1,2,8,*]

[7] These authors contributed equally: Hyunji Kang, Ah-reum Han, Aihua Zhang

*Correspondence: cjl@ibs.re.kr (C.J.L.), hm_kim@kaist.ac.kr (H.M.K.), sunchoi@ewha.ac.kr (S.C.)

This file includes:

- **Supplementary Figures 1-9 and Legends**
- **Supplementary Tables 1-5**

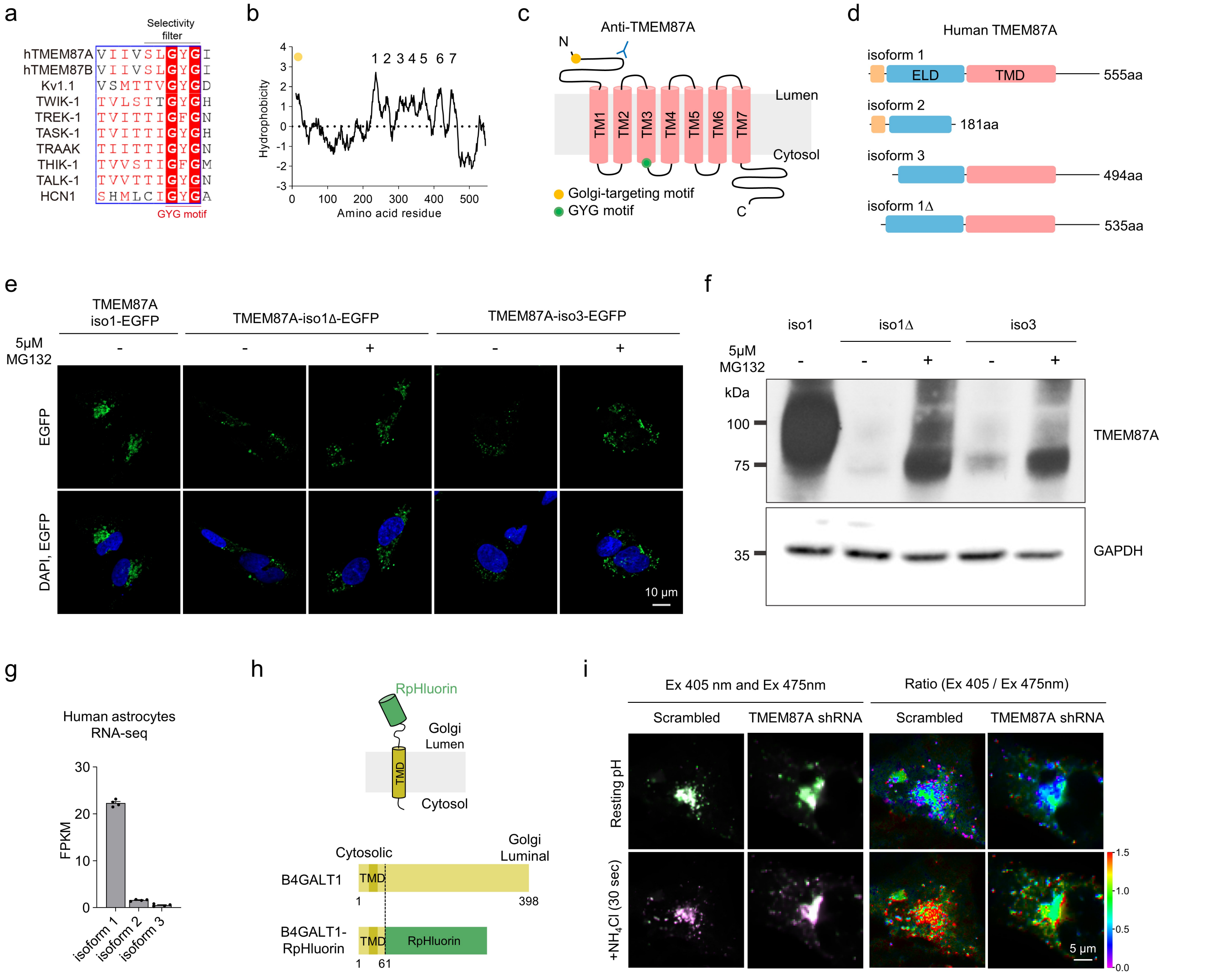

Supplementary Fig.1 | Golgi signal sequence in N-terminal of TMEM87A and Golgi pH regulation of TMEM87A

a, Partial sequence alignment for the selectivity filter of the K^+ channel family with hTMEM87A/B.

b, Hydrophobicity plot of hTMEM87A. Yellow circle indicates a predicted Golgi signal sequence site.

c, A predicted seven-transmembrane (TM) topology of TMEM87A within the Golgi lumen. Anti-TMEM87A indicates epitope for TMEM87A antibody. A green circle indicates a GYG motif sequence.

d, Proteins of human TMEM87A. ELD, extracellular domain; TMD, transmembrane domain; ss, signal sequence; isoform 1 Δss, ss deleted isoform 1.

e, Localization of C-terminal EGFP-tagged TMEM87A isoforms (iso1, isoΔ, iso3) in cultured human astrocytes in the absence or presence of 5μM MG132. Representative figures were obtained from three independent experiment.

f, Western blot analysis depicting the expression levels of EGFP-tagged TMEM87A isoforms (iso1, isoΔ, iso3) in cultured human astrocytes under conditions of both absence and presence of 5μM MG132. Representative figures were obtained from three independent experiment.

g, Transcriptome analysis of TMEM87A isoforms using RNA-seq in cultured human astrocytes (n=3cells).

h, Full length of B4GALT1 (left top). Constructs of B4GALT1 TMD-fused RpHluorin to target RpHluorin in the Golgi lumen (left bottom). Schematic diagram of used construct ‘B4GALT1-RpHluorin’ for measuring pH of Golgi lumen (right).

i. Representative merged fluorescence images illuminated with two excitation 405nm and 475nm with 50 mM NH_4Cl treat before and after in Scrambled (n=14cells) or TMEM87A shRNA (n=18cells) transfected cultured human astrocytes (left). Representative fluorescence ratio (ex 405nm/ex 475nm) images (right).

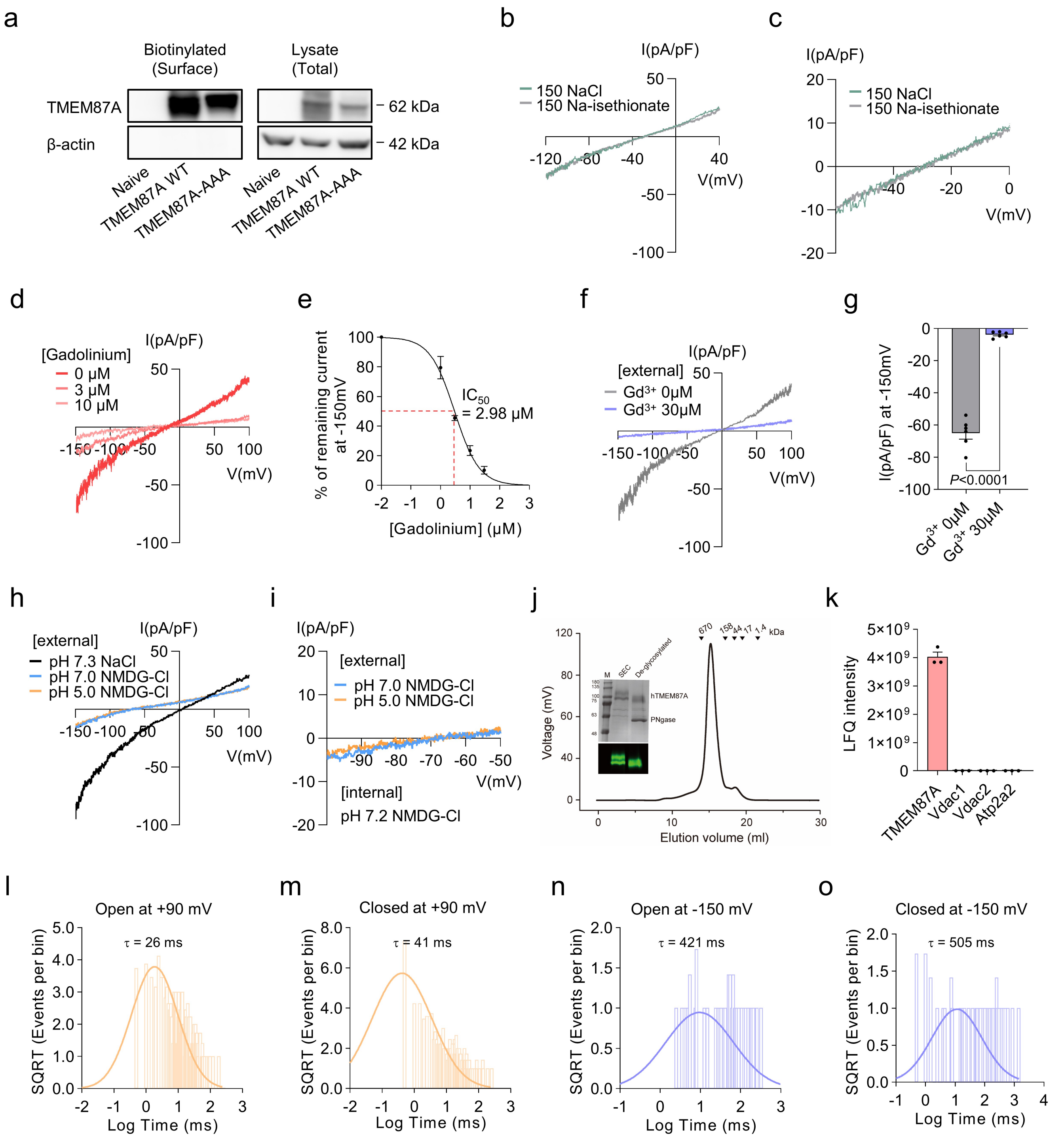

Supplementary Fig.2 | Electrophysiological characterization of TMEM87A

a, Surface biotinylation assay of TMEM87A WT and TMEM87A-AAA transfected cells blotted with TMEM87A antibody.

b, Representative I-V relationship from TMEM87A WT transfected cells under replacing the bath solution NaCl to Na-isethionate. The currents were corrected by liquid junction potential (LJP), respectively. (NaCl, green; Na-isethionate, gray; n=3cells).

c, Magnified trace for reversal potential from (b) (n=3cells).

d, Representative **I**-V relationship from TMEM87A WT transfected cell (n=5cells) with gadolinium in the bath solution.

e, Dose-response curve for percentage currents at -150 mV for gadolinium (0, 1, 3, 10, and 30 μM) (n=5cells).

f, Representative **I**-V relationship from mouse TMEM87A WT transfected cells (n=6cells) under bath solution without (Gd^{3+} 0 μM) or with 30 μM gadolinium (Gd^{3+} 30 μM).

g, Current densities measured at -150mV from (f) (n=6cells).

h, Representative **I**-V relationship from TMEM87A WT transfected cells under replacing the bath solution pH 7.3 NaCl to pH 7.0 and 5.0 NMDG-Cl. (pH 7.3 NaCl, black; pH 7.0 NMDG-Cl, blue; pH 5.0 NMDG-Cl, orange; n=3cells).

i, Magnified trace for reversal potential from (h) (n=3cells).

j, Size-exclusion chromatography (SEC) profile of hTMEM87A-EGFP-Twin-strep. Inset: SDS-PAGE analysis of hTMEM87A-EGFP-Twin-strep after PNGase F treatment (n=3hippocampal samples).

k, Bar graph of LFQ intensity of TMEM87A and other pore-forming ion channels involved in TMEM87A purified solution.

l,m, Dwell time histograms of TMEM87A single channel unitary current activities at +90 mV for open in **l** and closed in **m** states (n=3). Data are fitted with a log-normal distribution.

n,o, Dwell time histograms of TMEM87A single channel unitary current activities at -150 mV for open in **n** and closed in **o** states (n=3). Data are fitted with a log-normal distribution.

Data are presented as the mean ± SEM. Statistical analyses were performed using Mann-Whitney test in (g). Source data and exact p values are provided as a Source Data file.

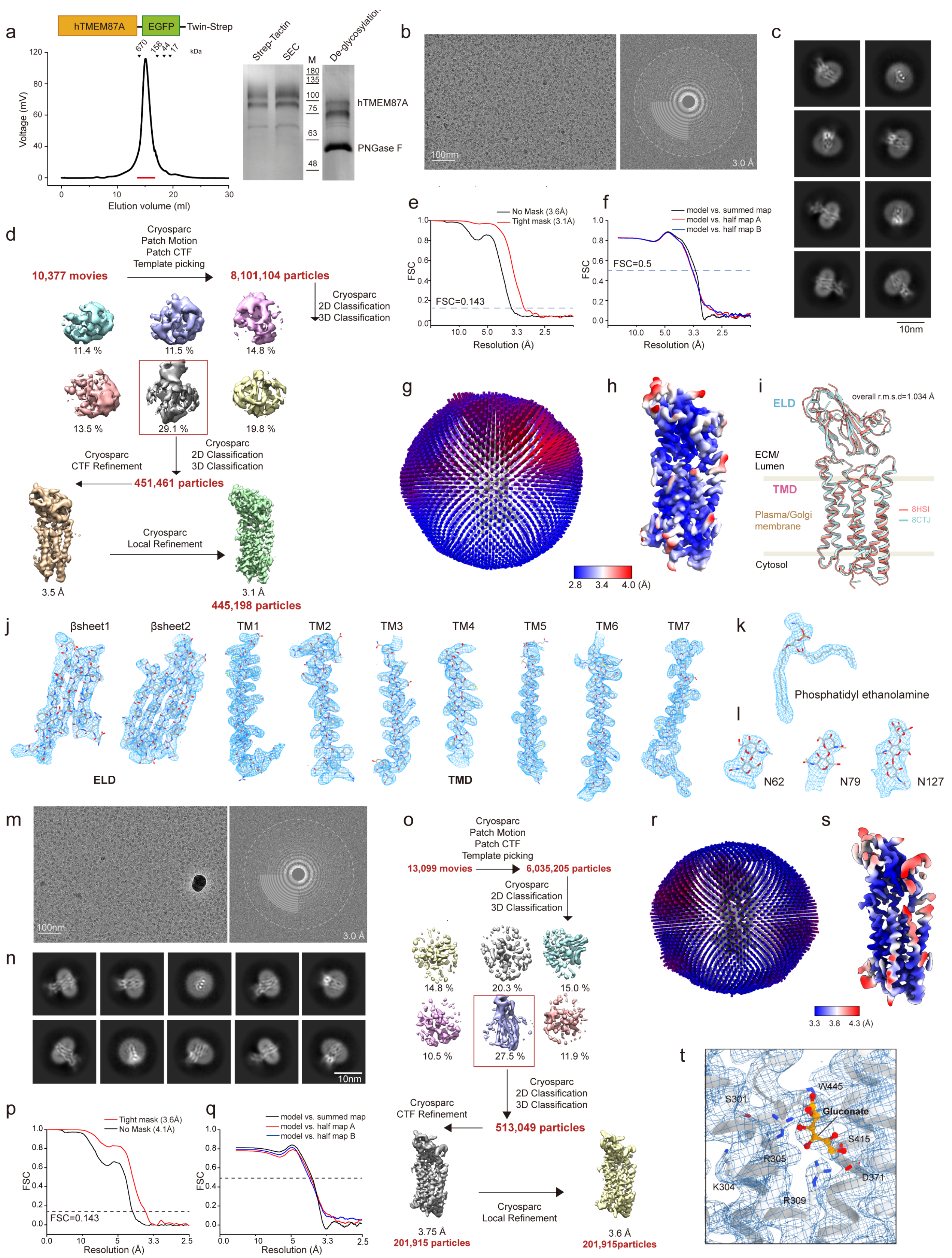

Supplementary Fig.3 | Purification and Cryo-EM analysis of hTMEM87A and hTMEM87A-Gluc

a, Expression construct and size-exclusion chromatography (SEC) profile of hTMEM87A-EGFP-Twin-strep (left). Full-length hTMEM87A (M1~E555) with a cleavable C-terminus EGFP-tag and a Twin-strep tag was expressed in Expi293F cells, solubilized with n-dodecyl β-D-maltoside (DDM) and cholesteryl hemisuccinate (CHS), and purified hTMEM87A by Strep-Tactin affinity purification and SEC in lauryl maltose neopentyl glycol (LMNG) and CHS. Pooled fractions for cryo-EM analysis are marked with the red bar. Coomassie blue stained SDS-PAGE gel of the elution fractions from Strep-Tactin affinity purification and the SEC peak fractions, respectively (middle). SDS-PAGE analysis of hTMEM87A-EGFP-Twin-strep after PNGase F treatment (right). Homogeneous peak fractions containing hTMEM87A-EGFP-Twin-strep protein (hTMEM87A) without proteolytic cleavage were used for structural studies.

b, Representative cryo-EM micrograph (left) and its Fourier transform (right).

c, Representative 2D class averages of hTMEM87A.

d, Data processing workflow of cryo-EM analysis of hTMEM87A.

e, Fourier shell correlation (FSC) curves between two independently refined half maps in cryoSPARC (left, resolution cutoff at FSC = 0.143)

f, FSC curves for cross-validation of a model (right, resolution cutoff at FSC = 0.5).

g, Euler angle distribution of all particles used in the final 3D reconstructions. The cylinder bars' height and color (from blue to red) are proportional to the number of particles in those views.

h, Final cryo-EM map of hTMEM87A colored with local resolution.

i, Comparison of two cryo-EM structures of hTMTM87A. Structure of hTMEM87A in detergent LMNG/CHS (light orange, our structure PDB:8HSI) and previously reported structure of hTMEM87A in Nanodisc (light blue, Hoel et al.'s PDB: 8CTJ) are superimposed. The overall architecture of the two structures is similar (overall r.m.s.d.=1.034Å).

j, Cryo-EM map and model from hTMEM87A. βsheet1 (β1, D38-S48; β3, N62-E72; β7, N204-P215, contour level = 0.142) and βsheet2 (β2, G49-F60; β4, L76-D88; β5, L117-Q126, β6, A180-S192, contour level = 0.129) of ELD. Seven helices of TMD (TM1, E222-L256, contour level = 0.101; TM2, R257-E288, contour level = 0.101; TM3, V290-V322, contour level = 0.101; TM4, V333-G354, contour level = 0.101; TM5, Q356-R393, contour level = 0.101; TM6, N394-I428, contour level = 0.101; and TM7, S433-P473, contour level = 0.101).

k, Cryo-EM map and model of the phosphatidylethanolamine (contour level = 0.101).

l, Cryo-EM map and model of three N-linked oligosaccharides (N62, contour level = 0.0805; N79, contour level = 0.0805; and N127, contour level = 0.0805).

m, Representative cryo-EM micrograph (left) and its Fourier transform (right) of the hTMEM87A-Gluc.

n, Representative 2D class averages of hTMEM87A-Gluc.

o, Data processing workflow of cryo-EM analysis of hTMEM87A-Gluc.

p, Fourier shell correlation (FSC) curves between two independently refined half maps in cryoSPARC (left, resolution cutoff at FSC = 0.143).

q, FSC curves for cross-validation of a model (right, resolution cutoff at FSC = 0.5).

r, Euler angle distribution of all particles used in the final 3D reconstructions. The cylinder bars' height and color (from blue to red) are proportional to the number of particles in those views.

s, Final cryo-EM map of hTMEM87A-Gluc colored with local resolution.

t, Cryo-EM map and model of hTMEM87A-Gluc in Glutamate-binding site (contour level = 0.125). Glutamate and interacting residues are shown as sticks and labeled.

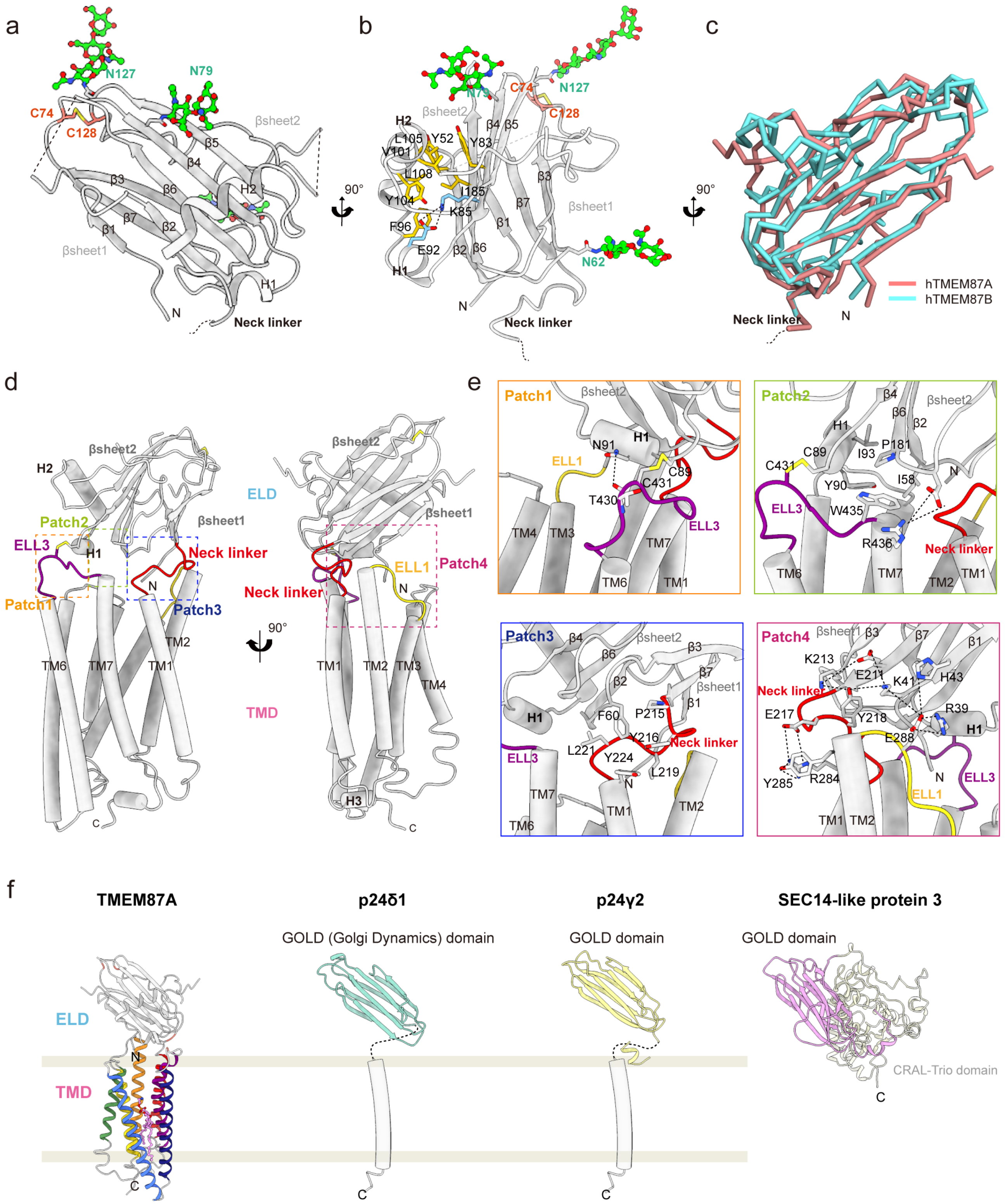

Supplementary Fig.4 | Structure of hTMEM87A ELD

a, Structure of hTMEM87A ELD. The structure is shown as a cartoon and colored in light gray. The ELD resembles a β-sandwich fold connected to the first TMD helix by a linker (termed a neck linker, G214-D223) and is composed of seven anti-parallel β-strands (β1, β3, β7 for βsheet1 and β2, β4, β5, β6 for βsheet2) and two short α-helices

(H1 and H2). The glycosylation site (N62, N79, and N127) and disulfide bridge (C74-128) are indicated. Two conserved cysteines (C74 and C128) form a disulfide bond that bridges a long loop between β5 and β6 to a neighboring loop between β3 and β4. Disulfide bridge (yellow) and N-linked glycans (green) are shown as sticks. Disordered regions were indicated as dashed lines.

b, Detail interactions of two short α-helices (H1 and H2) with the outer surface of βsheet2. Key interacting residues are displayed as sticks and labeled (ionic 5 interaction: sky blue, hydrophobic interactions: yellow). The H1 and H2 located between β4 and β5 of ELD interacts with the outer surface of βsheet2 through long-range hydrophobic networks (I93, F96, V101, Y104, L105, and L108 on H1 and H2; Y52, Y83, Y118, I183 and I185 on βsheet2) and electrostatic interaction (K85-E92). N-linked glycans (N62, N79, and N127) are shown as green sticks.

c, Superimposition of hTMEM87A ELD (salmon) with the hTMEM87B ELD (cyan, NP_116213.1, E45-S212), whose structure is predicted by Alphafold. The calculated Cα root mean square deviation (R.M.S.D) is 2.24.

d, Interaction interfaces between ELD and TMD. Four interaction patches are indicated by dotted box (patch1, orange; patch2, lime; patch3, blue; and patch4, magenta). Loops involving interface interactions were highlighted (Neck linker: red, ELL1: yellow, and ELL3: purple).

e, Close-up views of each interaction interface in (d). Key interacting residues are shown as sticks and labeled. Hydrogen and ionic bonds are indicated as a dashed line.

f, Structural comparison of the hTMEM87A ELD with other Golgi dynamics (GOLD) domain-containing proteins [p24δ1 (PDB: 5AZX, light teal), p24γ2 (PDB:5GU5, pale yellow), and SEC14-like protein 3 (PDB: 4UYB, light pink).

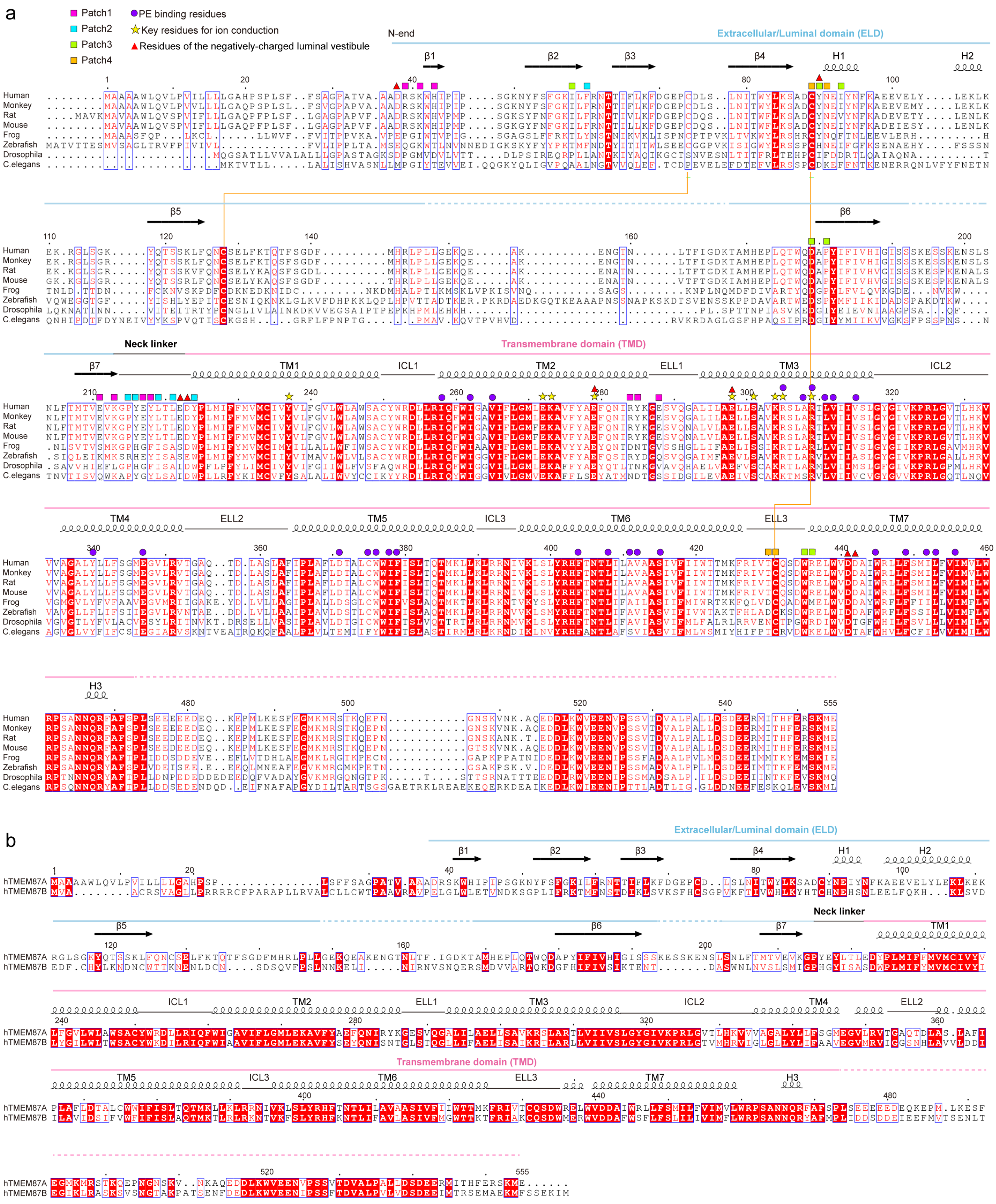

Supplementary Fig.5 | Multiple sequence alignment of TMEM87A

a, Amino acid sequence alignment of human (*Homo sapiens*, NP_056312.2), monkey (*Chlorocebus sabaeus,*

XP_008015094.1), rat (*Rattus norvegicus*, XP_038962452.1), mouse (*Mus musculus*, NP_776095.2), frog (*Xenopus tropicalis*, NP_001016057.1), Zebrafish (*Danio rerio*, NP_001082853.2), Fruit fly (*Drosophila melanogaster*, NP_608612.3), Roundworm (*Caenorhabditis elegans*, NP_508729.2). TMEM87A orthologs are obtained by ortholog search. Domains (ELD, TMD, N-end, and neck linker) and secondary structure elements (arrows for β stands and helices for α-helices) are displayed above the alignment. Squares indicate interacting residues at the interface between ELD and TMD (patch1: orange, patch2: green, patch3: cyan, and patch4: magenta). Purple circles indicate interacting residues with phosphatidyl ethanolamine (PE). Yellow stars indicate key residues involved in ion conduction. Red triangles indicate residues consisting of the negatively-charged luminal vestibule. Disulfide bonds (orange lines) are also indicated. Red boxes indicate perfect sequence conservation, whereas blue-lined boxes show residues with >70% similarity based on physicochemical properties.

b, Amino acid sequence alignment of hTMEM87A and hTMEM87B (NP_116213.1). Domains (ELD, TMD, N-end, and neck linker), secondary structure elements (arrows for β stands and helices for α-helices), and disulfide bonds (orange lines) are displayed. Red boxes indicate perfect sequence conservation, whereas blue-lined boxes show residues with >70% similarity based on physicochemical properties.

The sequence alignment was created using Clustal Omega (https://www.ebi.ac.uk/Tools/msa/clustalo/) and EsPript 3.0 (https://espript.ibcp.fr/ESPript/ESPript/).

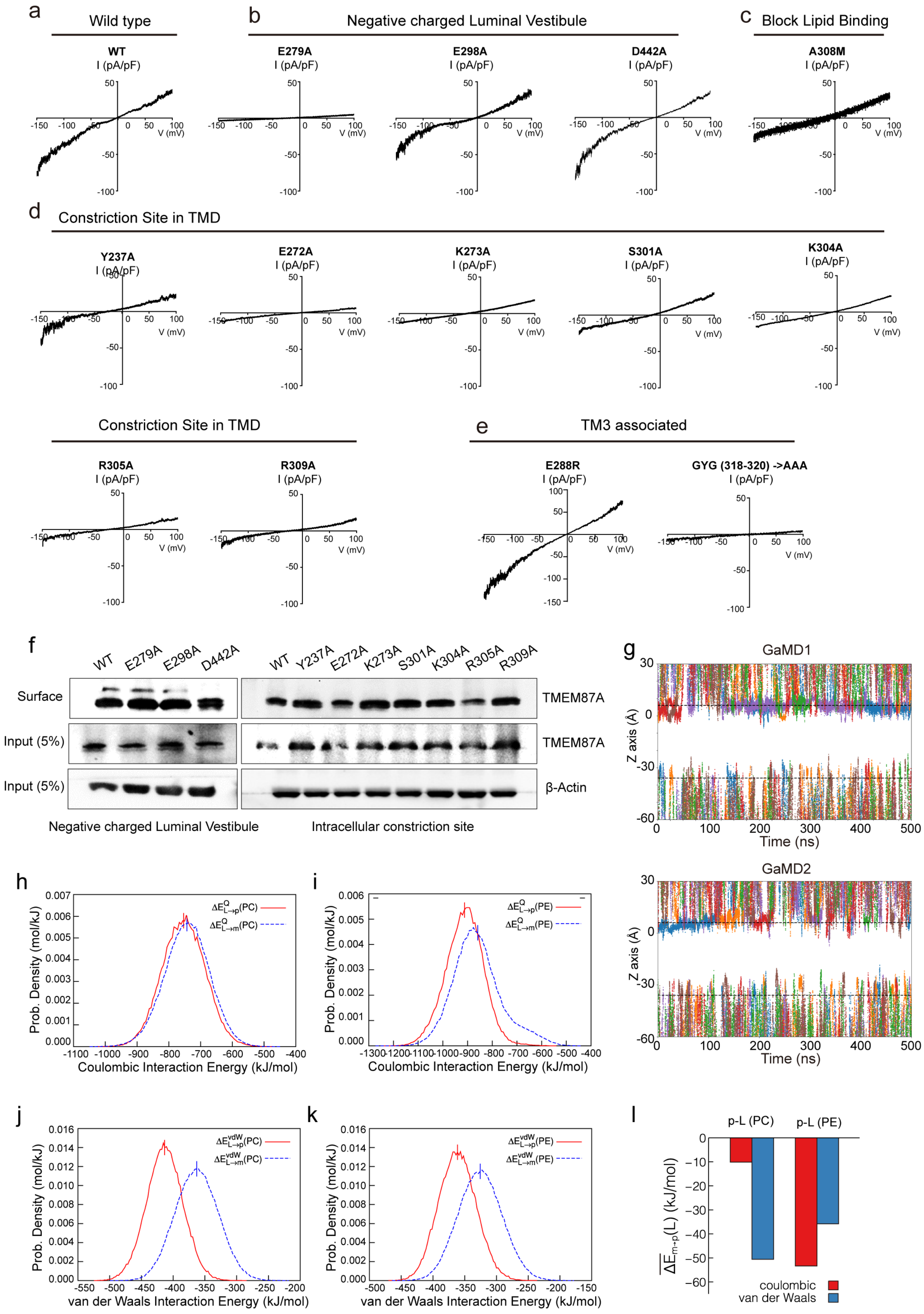

a
Wild type
WT
b
Negative charged Luminal Vestibule
E279A
E298A
D442A
c
Block Lipid Binding
A308M
d
Constriction Site in TMD
Y237A
E272A
K273A
S301A
K304A
Constriction Site in TMD
R305A
R309A
e
TM3 associated
E288R
GYG (318-320) ->AAA
I (pA/pF)
V (mV)
f
WT E279A E298A D442A
WT Y237A E272A K273A S301A K304A R305A R309A
Surface
Input (5%)
Input (5%)
TMEM87A
TMEM87A
β-Actin
Negative charged Luminal Vestibule
Intracellular constriction site
g
GaMD1
GaMD2
Z axis (Å)
Time (ns)
h
i
Prob. Density (mol/kJ)
Coulombic Interaction Energy (kJ/mol)
j
k
van der Waals Interaction Energy (kJ/mol)
l
p-L (PC)
p-L (PE)
coulombic
van der Waals

Supplementary Fig.6 | Electrophysiology of hTMEM87A WT and mutants, and molecular dynamics simulations

a-e, Representative current-voltage (I-V) curves obtained in whole-cell configuration of hTMEM87A WT and its mutants. The currents were recorded with voltage-clamp ramp protocol descending from +100 mV to -150 mV.

a, Representative current-voltage (I-V) curves of hTMEM87A WT.

b, Representative I-V relationship of hTMEM87A mutants (E279A, E298A, and D442A) for NLV.

c, Representative I-V relationship of hTMEM87A mutants (A308M) for blocking lipid binding.

d, Representative I-V relationship of hTMEM87A mutants (Y237A, E272A, K273A, S301A, K304A, R305A, and R309A) for constriction site in TMD

e, Representative I-V relationship of hTMEM87A mutants (E288R and AAA) for TM3 associated.

f, Membrane expression of hTMEM87A WT and its mutant measured by surface biotinylation assay. HEK293A cells were transfected with hTMEM87A WT or its mutants (E279A, E298A, D442A, Y237A, E272A, K273A, S301A, K304A, R305A, R309A, S415F, and S415W), treated with biotin to isolate surface hTMEM87A proteins. Proteins were immunoblotted with TMEM87A antibodies (Novus). These results indicate that all of hTMEM87A mutants are expressed in the plasma membrane at a similar level.

g, Representation of the Z coordinates of the K^+ atoms that bind in the negatively-charged luminal vestibule (NLV) along the GaMD simulations. The Z coordinates of the K^+ atoms from each binding event are indicated by different colored dots. The black dashed lines correspond to the upper and lower boundaries of the lipid bilayer membrane calculated as the averaged Z coordinates of the nitrogen atoms of the PE and PC molecules. Note that for the GaMD simulation 3 none binding event is explored, while for GaMD simulation 1 and 2 we explore 6 binding events each. When the binding takes place, the K^+ explores the NLV region (ca. 0-10 Z axis values) for a relatively long period of time.

h-k, The distributions of coulombic and van der Waals interaction energies for the PE/PC binding with hTMEM87A (solid lines) and for the PE/PC binding in a mixed lipid bilayer membrane (PC: PE=3:1) (dashed lines). The average interaction energies are marked by the vertical lines. Five 1 μs trajectories were simulated in the solvated system ($S_{p\text{-}L}$), where hTMEM87A structures containing either lipid (PC or PE) bound to the TMD cavity were embedded in a simple Golgi model membrane (PC:PE=3:1). The results showed that the van der Waals interaction energy of PC is increased due to the additional methyl groups, while its coulombic interaction energy decreased because of their screening effect.

l, Comparing average coulombic/van der Waals interaction energies of PE/PC transferring from membrane to protein. The transferring interaction energies are calculated as the differences between the average interaction energies in the b-e plots. The binding free energies in the main text can be calculated according to the LIE formula, with 0.5 and 0.16 being the coefficients for the coulombic/van der Waals contributions, respectively. Source data are provided as a Source Data file.

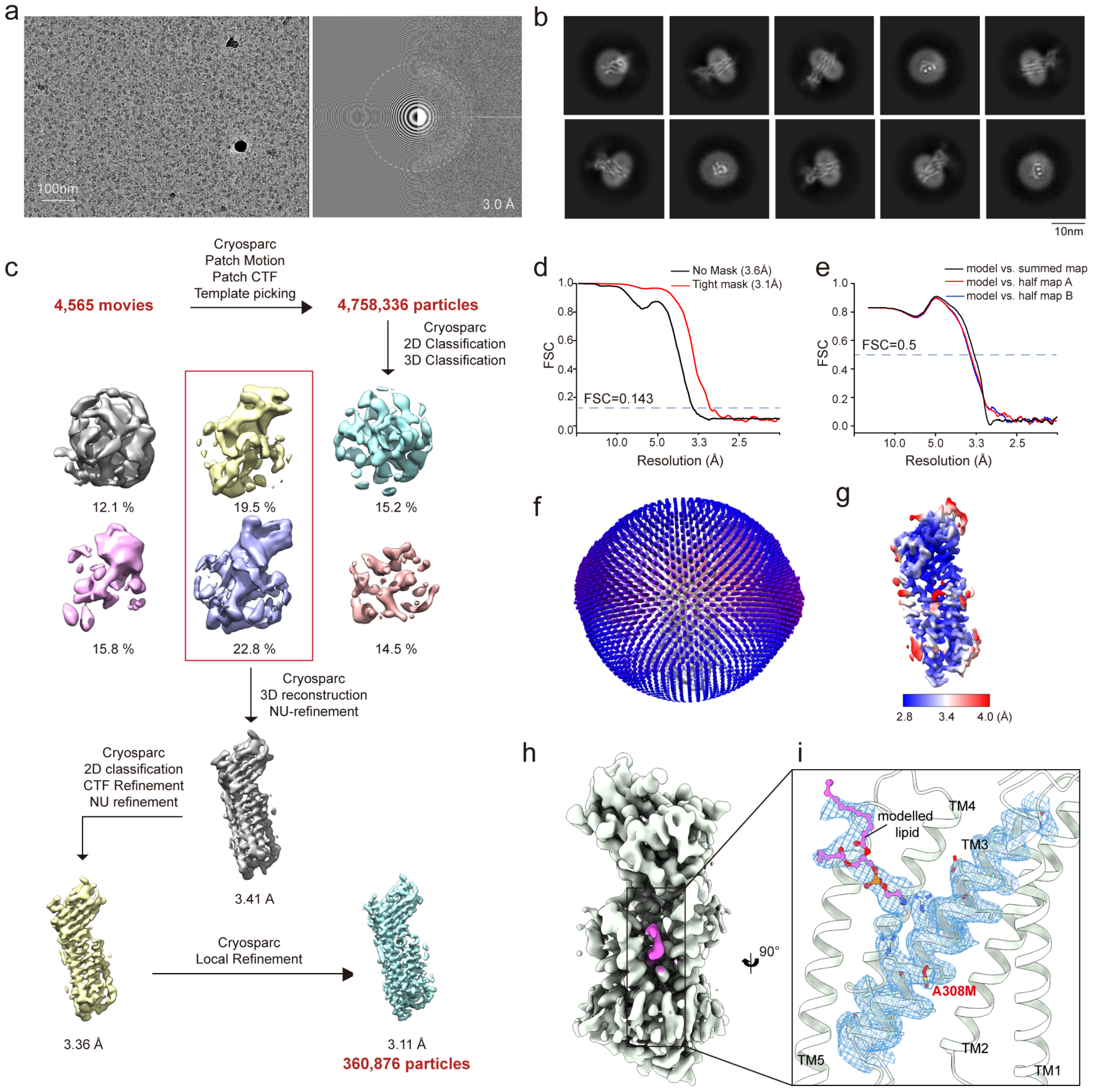

Supplementary Fig.7 | Cryo-EM analysis of hTMEM87A A308M

a, Representative cryo-EM micrograph (left) and its Fourier transform (right) of the hTMEM87A A308M.

b, Representative 2D class averages of hTMEM87A-A308M.

c, Data processing workflow of cryo-EM analysis of hTMEM87A-A308M.

d, Fourier shell correlation (FSC) curves between two independently refined half maps in cryoSPARC (left, resolution cutoff at FSC = 0.143).

e, FSC curves for cross-validation of a model (right, resolution cutoff at FSC = 0.5).

f, Euler angle distribution of all particles used in the final 3D reconstructions. The cylinder bars' height and color (from blue to red) are proportional to the number of particles in those views.

g, Final cryo-EM map of hTMEM87A A308M colored with local resolution.

h, Cryo-EM map and model of hTMEM87A A308M. Overall cryo-EM density map of hTMEM87A A308M (contour level = 0.101) is displayed with the same view in Fig.3g. The density of the lipid is shown in pink.

i, Model fit to the cryo-EM map in the lipid binding site of hTMEM87A A308M. Densities of lipid and TM3 are shown (contour level = 0.085). A modeled lipid is shown as pink sticks. The A308M mutation is highlighted in red.

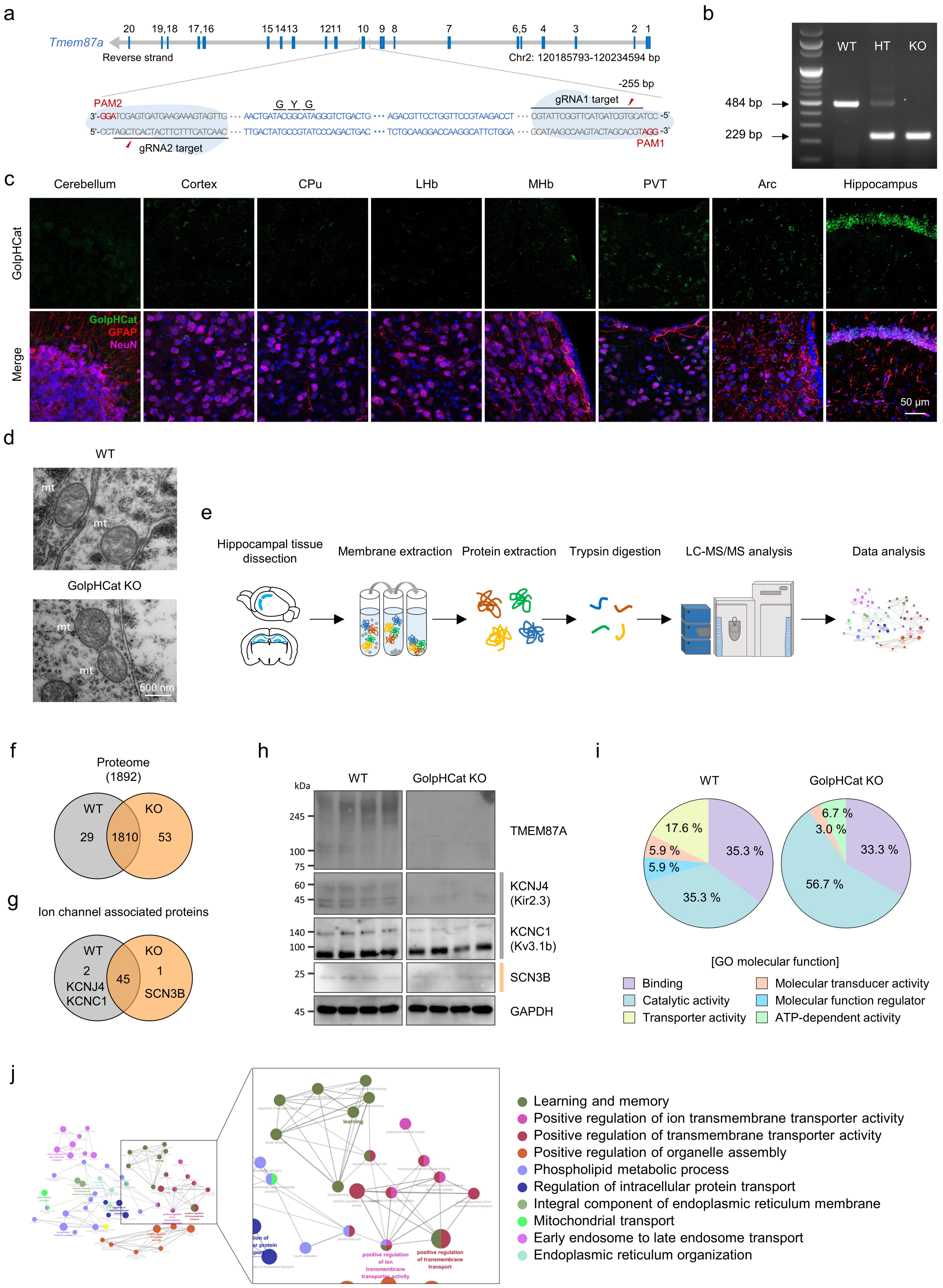

Supplementary Fig.8 | Generation of GolpHCat KO mice and altered biological functions in GolpHCat KO mice

a, Schematic diagram of *Tmem87a* genetic locus on the reverse strand of mouse chromosome 2. Exons are indicated with a blue box with a number, and introns are indicated by a gray line (top). The target genomic locus of two gRNAs on introns 9 and 10 are noted in blue, with the protospacer adjacent motif (PAM) in red (bottom).

b, Representative PCR Genotyping result of wild-type, GolpHCat heterozygote and homozygote mice. Representative figures were obtained from five independent experiment.

c, GolpHCat expression in various brain regions including cerebellum, cortex, caudate putamen (CPu), lateral habenula (LHb), medial habenula (MHb), paraventricular thalamic nucleus (PVT), and arcuate hypothalamus (Arc). Representative figures were obtained from three independent experiment.

d, Representative TEM images of the mitochondria in WT and GolpHCat KO mice (n=4cells from 3mice).

e, Workflow of proteomics analysis with hippocampus sample.

f, Venn diagram comparison of identified proteins in hippocampus of WT (n=3) versus GolpHCat KO (n=3).

g, Venn diagram comparison of identified ion channel associated proteins in hippocampus of WT versus GolpHCat KO.

h, Western blotting of GolpHCat and identified ion channel associated proteins in hippocampus of WT (n=4) versus GolpHCat KO (n=4)

i, Gene Ontology (GO) term analysis with the differentially expressed proteins of WT and GolpHCat KO. Pie chart depicting the percentage of involvement of identified proteins and their molecular functions.

j, Network clusters performed with differentially expressed proteins of WT and GolpHCat KO in Cytoscape using ClueGO and CluePedia. The size of the nodes corresponds to the number of proteins associated to a term.

Source data are provided as a Source Data file.

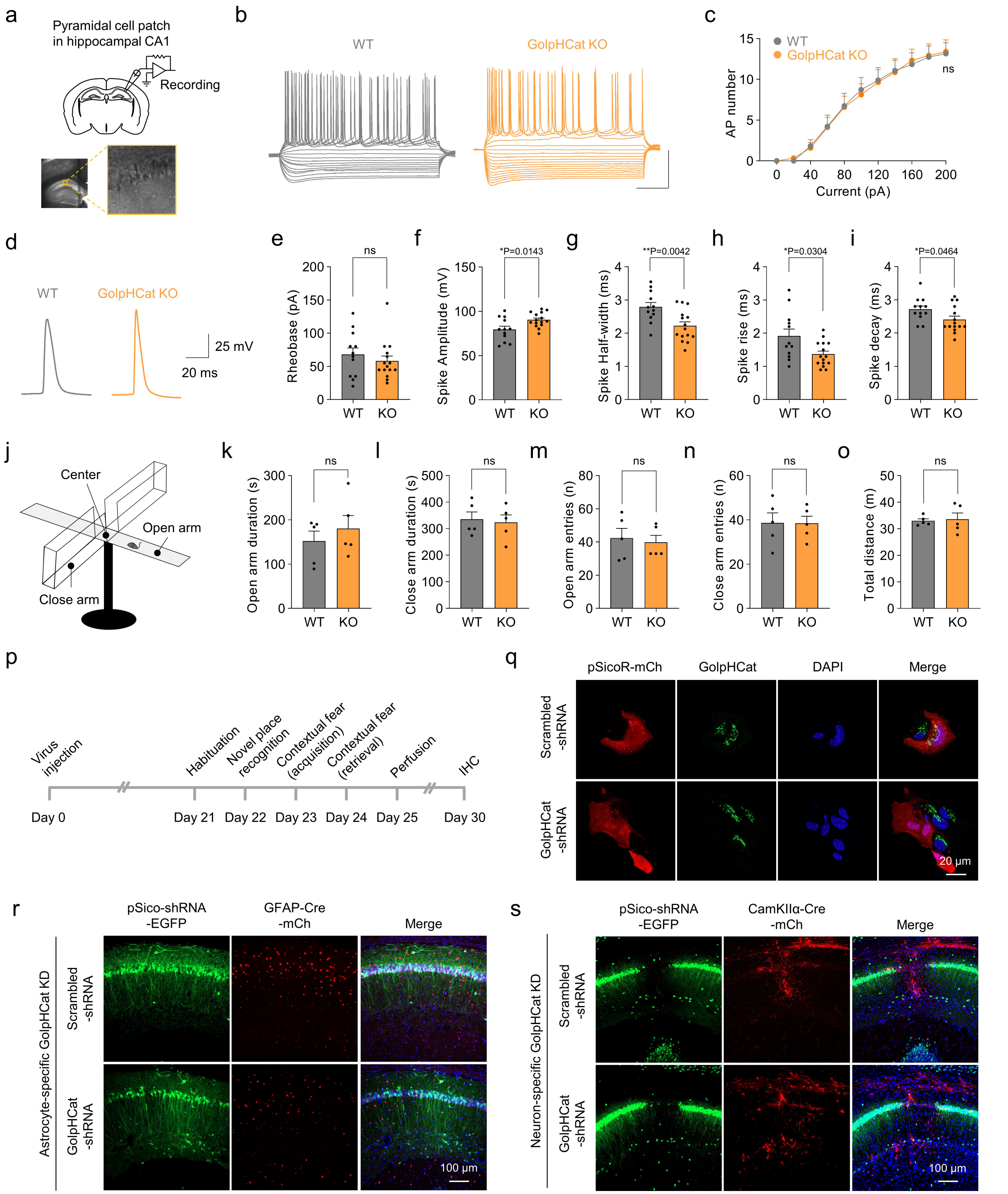

Supplementary Fig.9 | Intrinsic properties of TMEM87A KO and cell-type specific gene silencing for behavioral tests

a, Schematic diagram of a slice patch from WT and GolpHCat KO mice (top) and magnified differential interference contrast (DIC) image of a whole-cell patch-clamped CA1 pyramidal neuron in the hippocampus (bottom).

b, Representative action potential traces in the CA1 pyramidal neurons in WT (n=12 from 3 mice) and GolpHCat KO (n=15 from 3 mice) mice. Scale bar: 50 mV and 200 ms.

c, Spike (action potential) number under the depolarizing current step protocol (from 0 pA to +200 pA, 20 pA step) in the CA1 pyramidal neurons in WT (n=12 from 3 mice) and GolpHCat KO (n=15 from 3 mice) mice.

d, Representative single action potential traces of WT (n=12 from 3 mice) and GolpHCat KO (n=15 from 3 mice) mice.

e-I, Bar graph of rheobase (e), spike amplitude (f), spike half-width (g), spike rise (h), and spike decay (i) of WT (n=12 from 3 mice) and GolpHCat KO (n=15 from 3 mice) mice.

j, Schematic diagram of the elevated plus maze (EPM).

k-o, Bar graph of open arm duration (k), close arm duration (l), open arm entries (m), close arm entries (n), and total distance (o) in elevated plus maze test of WT (n=5) and GolpHCat KO (n=5) mice.

p, Experimental timeline for virus injection, behavior tests, and IHC.

q, Immunostaining of GolpHCat in the scrambled shRNA or TMEM87A shRNA microporated cultured hippocampal astrocytes for confirming the efficiency of GolpHCat shRNA. Representative figures were obtained from three independent experiment.

r, Fluorescence images in AAV-GFAP-cre-mCh + Lenti-pSico-Scrambled (n=9mice)/ GolpHCat shRNA (n=9mice)-EGFP virus-injected mice.

s, Fluorescence images in AAV-CamKII-cre-mCh + Lenti-pSico-Scrambled (n=8mice)/ GolpHCat shRNA (n=10mice)-EGFP virus-injected mice.

Data are presented as the mean ± SEM. Statistical analyses were performed using two-way ANOVA followed by Holm-Šídák's multiple comparisons test in (c), Mann-Whitney test in (e and k-o), two-tailed unpaired t-test in (f), (g), and (i), unpaired t-test with Welch's correction (h). Source data and exact p values are provided as a Source Data file.

Supplementary Table 1. Mass spectrometry of the purified EGFP-tagged TMEM87A solution

Protein names	Sequence coverage [%]	Unique peptides	LFQ intensity (Sample 1)	LFQ intensity (Sample 2)	LFQ intensity (Sample 3)	% of LFQ intensity relative to TMEM87A (Sample 1)	% of LFQ intensity relative to TMEM87A (Sample 2)	% of LFQ intensity relative to TMEM87A (Sample 3)
Transmembrane protein 87A	38.2	17	3843600000	3841300000	4381300000	100	100	100
Calnexin	29.1	18	308190000	311120000	325550000	8.018264	8.099341	7.430443
Heat shock 70 kDa protein 1A;Heat shock 70 kDa protein 1B	49.1	11	304680000	278460000	305920000	7.926943	7.249108	6.982402
Heat shock cognate 71 kDa protein	59.1	23	222450000	207420000	240420000	5.787543	5.399734	5.487412
Ubiquitin-60S ribosomal protein L40;	35.2	4	149390000	137980000	157380000	3.886721	3.592013	3.592085
Heat shock 70 kDa protein 1-like	24	2	104070000	97057000	103330000	2.707618	2.526671	2.358432
Poly(ADP-ribose) glycohydrolase	1.1	1	96131000	130210000	108280000	2.501067	3.389738	2.471413
Vascular endothelial growth factor C	8	3	46361000	45273000	46274000	1.206187	1.178585	1.056171
Vascular endothelial growth factor receptor 3	3.2	4	38250000	34194000	35565000	0.995161	0.890167	0.811745
Tubulin alpha-1C chain;Tubulin alpha-8 chain	41.4	1	15071000	14847000	15578000	0.392106	0.38651	0.355557
VIP36-like protein	18.7	6	14925000	14209000	16089000	0.388308	0.369901	0.36722
Histone H4	29.1	3	8314500	8149600	10355000	0.216321	0.212157	0.236345
78 kDa glucose-regulated protein	40	18	8171600	7793100	8438600	0.212603	0.202877	0.192605
Calmegin	3.6	1	7736800	6473500	8605100	0.20129	0.168524	0.196405
Tubulin beta-5 chain;Tubulin beta-2B chain;Tubulin beta-2A chain	41.4	4	5992500	6312500	6596400	0.155909	0.164332	0.150558
Vesicular integral-membrane protein VIP36	17.9	5	5978200	6268600	6919500	0.155536	0.16319	0.157933
Histone H2A type 1	26.9	3	5627100	5018100	5529900	0.146402	0.130635	0.126216
Histone H2B type 1	14.3	2	4563300	4210100	4814100	0.118725	0.109601	0.109878
Stress-70 protein, mitochondrial	34.5	16	4180400	3939700	4417700	0.108763	0.102562	0.100831
Voltage-dependent anion-selective channel protein 1	26	5	3393600	3137200	3168500	0.088292	0.08167	0.072319
Voltage-dependent anion-selective channel protein 2	28.5	6	3028300	2867600	3151500	0.078788	0.074652	0.071931
RuvB-like 1	30.5	10	2249400	2001800	2228600	0.058523	0.052113	0.050866
Acetyl-CoA carboxylase 1;Biotin carboxylase	15.3	25	2171600	2022800	2352700	0.056499	0.052659	0.053699
Actin, cytoplasmic 2	11.5	3	2103500	1864400	2185700	0.054727	0.048536	0.049887
Membrane-associated progesterone receptor component 1 and 2	8.7	2	1859200	1574900	1821700	0.048371	0.040999	0.041579
Beta-1,4-glucuronyltransferase 1	19.5	6	1828500	1718300	1618500	0.047573	0.044732	0.036941
Hemoglobin subunit alpha;Hemoglobin subunit zeta	13.4	1	1803100	1513500	1575700	0.046912	0.039401	0.035964
60S ribosomal protein L23	14.3	1	1613000	2092500	2110200	0.041966	0.054474	0.048164
Propionyl-CoA carboxylase beta chain, mitochondrial	15.3	6	1550000	1497200	1663700	0.040327	0.038976	0.037973
RuvB-like 2	14.7	6	1528000	1442000	1599800	0.039754	0.037539	0.036514
S-phase kinase-associated protein 1	29.4	3	1301300	1205200	1219500	0.033856	0.031375	0.027834
Ras-related protein Rab-7a	28.5	5	1201500	1227600	1339700	0.03126	0.031958	0.030578
Sarcoplasmic/endoplasmic reticulum calcium ATPase 2	6.3	4	1185800	1168500	1327300	0.030851	0.030419	0.030295
Transmembrane protein 43	10.8	2	1092400	1086200	788910	0.028421	0.028277	0.018006
Ras-related protein Rab-6B;Ras-related protein Rab-6A	10.6	1	1011400	985770	1188300	0.026314	0.025662	0.027122
Maternal embryonic leucine zipper kinase	1.7	1	952870	0	1004300	0.024791	0	0.022922
Ras-related protein Rab-2A;Ras-related protein Rab-2B	24.1	4	943340	827780	920200	0.024543	0.021549	0.021003
Poly(rC)-binding protein 2;Poly(rC)-binding protein 3	10.5	3	915720	839080	911690	0.023825	0.021844	0.020809
Protein FAM3A	11.3	2	873680	827680	933800	0.022731	0.021547	0.021313
Synaptojanin-2-binding protein	24.8	3	841120	827790	789730	0.021884	0.02155	0.018025
Neutral alpha-glucosidase AB	2.2	2	787110	772720	820150	0.020478	0.020116	0.018719
Endoplasmin	3.7	2	728580	777130	800330	0.018956	0.020231	0.018267
Pyruvate carboxylase, mitochondrial	4.3	3	695950	614520	788160	0.018107	0.015998	0.017989

Supplementary Table 2. Cryo-EM data collection, refinement, and validation statistics

	#1 hTMEM87A (EMDB-34998) (PDB-8HSI)	#2 hTMEM87A-Gluc (EMDB-35017) (PDB-8HTT)	#2 hTMEM87A-A308M (EMDB-37069) (PDB-8KB4)
**Data collection and processing**			
Magnification	105,000x	105,000x	105,000x
Voltage (kV)	300	300	300
Electron exposure (e–/Å^2)	67.72	68.1	67.7
Defocus range (μm)	-0.8—1.9	-0.8—1.9	-0.7—1.7
Pixel size (Å)	0.849	0.849	0.848
Symmetry imposed	C1	C1	C1
Initial particle images (no.)	8,101,104	6,035,205	4,758,336
Final particle images (no.)	445,198	201,915	360,876
Map resolution (Å)	3.14	3.6	3.12
FSC threshold	0.143	0.143	0.143
Map resolution range (Å)			
**Refinement**			
Initial model used (PDB code)	-	8HSI	8HSI
Model resolution (Å)			
FSC threshold	0/0.143/0.5	0/0.143/0.5	0/0.143/0.5
Model resolution range (Å)	3.0/3.1/3.3	3.4/3.5/3.9	3.0/3.1/3.4
Map sharpening *B* factor (Å^2)	40.0	65.00	35.0
Model composition			
Non-hydrogen atoms	-	-	
Protein residues	405	389	402
Ligands	BMA:1, NAG:6, CLR:2, L9Q:1	BMA:1, NAG:6, GCO:1	NAG:3, 65I:1
B factors (Å^2)			
Protein	122.92	163.62	110.63
Ligand	119.42	217.22	155.27
R.m.s. deviations			
Bond lengths (Å)	0.028	0.003	0.004
Bond angles (°)	0.880	0.713	0.585
Validation			
MolProbity score	2.05	2.11	2.18
Clashscore	14.83	18.66	18.76
Poor rotamers (%)	0.0	0.0	0.00
Ramachandran plot			
Favored (%)	94.49	95.04	93.94
Allowed (%)	5.51	4.96	6.06
Disallowed (%)	0.0	0.0	0.0

Supplementary Table 3. Differentially expressed proteins found in WT and GolpHCat KO using mass spectrometry

**Differentially expressed proteins in WT**
Lysocardiolipin acyltransferase 1
Aladin
Envoplakin
Rootletin
Protocadherin gamma-A4
Sodium-dependent phosphate transporter 2
Long-chain specific acyl-CoA dehydrogenase, mitochondrial
Endophilin-B1
Diacylglycerol kinase epsilon
Phosphoenolpyruvate carboxykinase [GTP], mitochondrial
Signal peptidase complex subunit 2
Metaxin-2
Copine-2
Adenylate kinase isoenzyme 1
Potassium voltage-gated channel subfamily C member 1
Paraplegin
Guanylate cyclase D
Rho GTPase-activating protein 32
Inward rectifier potassium channel 4
Polypeptide N-acetylgalactosaminyltransferase 16
Carboxypeptidase D
A-kinase anchor protein 1, mitochondrial
Calcium signal-modulating cyclophilin ligand
Kelch repeat and BTB domain-containing protein 11
Glypican-4;Secreted glypican-4;Glypican-6;Secreted glypican-6
Annexin A1
Ig mu chain C region
Abelson tyrosine-protein kinase 2
Zinc finger ZZ-type and EF-hand domain-containing protein 1

**Differentially expressed proteins in GolpHCat KO**
TNF receptor-associated factor 3
Ubiquitin-conjugating enzyme E2 D3;Ubiquitin-conjugating enzyme E2 D2;Ubiquitin-conjugating enzyme E2 D2B
Transmembrane protein 163
Transmembrane anterior posterior transformation protein 1
Selenoprotein T
Serine incorporator 1
Aspartyl/asparaginyl beta-hydroxylase
Coiled-coil domain-containing protein 132
Acyl-coenzyme A thioesterase 13;Acyl-coenzyme A thioesterase 13, N-terminally processed
DnaJ homolog subfamily A member 2
Multidrug resistance protein 1A;Multidrug resistance protein 1B
Rab-interacting lysosomal protein
Serine/threonine-protein kinase PAK 1
RNA-binding protein 14

Sodium channel subunit beta-3
Mitochondrial import inner membrane translocase subunit TIM16
Serum paraoxonase/arylesterase 2
ATPase Asna1
CAAX prenyl protease 1 homolog
Spermatogenesis-defective protein 39 homolog
Type 1 phosphatidylinositol 4,5-bisphosphate 4-phosphatase
Neuron-specific protein family member 2
Phytanoyl-CoA hydroxylase-interacting protein
ADP-ribosylation factor-like protein 6-interacting protein 1
NADH dehydrogenase [ubiquinone] iron-sulfur protein 6, mitochondrial
Tumor protein D52
Leukocyte elastase inhibitor A
Sepiapterin reductase
Serine/threonine-protein kinase B-raf;RAF proto-oncogene serine/threonine-protein kinase
Calcium-independent phospholipase A2-gamma
GTP-binding protein Di-Ras1
Inactive phospholipase C-like protein 2
TOM1-like protein 2
Presqualene diphosphate phosphatase
Transmembrane 9 superfamily member 4
Kv channel-interacting protein 4;Kv channel-interacting protein 1
Lipid phosphate phosphatase-related protein type 3
Heterogeneous nuclear ribonucleoprotein A1;Heterogeneous nuclear ribonucleoprotein A1, N-terminally processed
Constitutive coactivator of PPAR-gamma-like protein 1
Multifunctional protein ADE2;Phosphoribosylaminoimidazole-succinocarboxamide synthase;Phosphoribosylaminoimidazole carboxylase
Probable ATP-dependent RNA helicase DDX17
Sorting nexin-2
Moesin
MAP/microtubule affinity-regulating kinase 4
Alpha-(1,6)-fucosyltransferase
Amyloid beta A4 precursor protein-binding family B member 1
Protein S100-B
Plexin-A2
Protein kinase C delta type;Protein kinase C delta type regulatory subunit;Protein kinase C delta type catalytic subunit
Microtubule-associated serine/threonine-protein kinase 3
Huntingtin
Isoleucine--tRNA ligase, cytoplasmic
Diacylglycerol kinase gamma

Supplementary Table 4. Constructs for recombinant protein expression

Construct	Expression vector	Information
hTMEM87A-WT-GFP-Strep	pcDNA3.4 TOPO	HindIII-hTMEM87A-WT (M1-E555)-TEV site-3C site-EGFP-)-thrombin site-Twin-Strep-tag-stop-XhoI
hTMEM87A-WT	pIRES2-DsRed	SalI-hTMEM87A-WT (M1-E555)-BamHI
hTMEM87A-AAA (GYG318~320AAA)	pIRES2-DsRed	SalI-hTMEM87A-AAA (M1-E555)-BamHI
hTMEM87A-Y237A	pIRES2-DsRed	SalI-hTMEM87A-Y237A (M1-E555)-BamHI
hTMEM87A-E272A	pIRES2-DsRed	SalI-hTMEM87A-E272A (M1-E555)-BamHI
hTMEM87A-K273A	pIRES2-DsRed	SalI-hTMEM87A-K273A (M1-E555)-BamHI
hTMEM87A-E279A	pIRES2-DsRed	SalI-hTMEM87A-E279A (M1-E555)-BamHI
hTMEM87A-E298A	pIRES2-DsRed	SalI-hTMEM87A-E298A (M1-E555)-BamHI
hTMEM87A-S301A	pIRES2-DsRed	SalI-hTMEM87A-S301A (M1-E555)-BamHI
hTMEM87A-K304A	pIRES2-DsRed	SalI-hTMEM87A-K304A (M1-E555)-BamHI
hTMEM87A-R305A	pIRES2-DsRed	SalI-hTMEM87A-R305A (M1-E555)-BamHI
hTMEM87A-R309A	pIRES2-DsRed	SalI-hTMEM87A-R309A (M1-E555)-BamHI
hTMEM87A-A308M	pIRES2-DsRed	SalI-hTMEM87A- A308M (M1-E555)-BamHI
hTMEM87A-R257A/R325A	pIRES2-DsRed	SalI-hTMEM87A- R257A/R325A (M1-E555)-BamHI
hTMEM87A-E288R	pIRES2-DsRed	SalI-hTMEM87A- E288R (M1-E555)-BamHI
hTMEM87A-D442A	pIRES2-DsRed	SalI-hTMEM87A-D442A (M1-E555)-BamHI

Supplementary Table 5. Primer sequences used for cloning

Primer	Sequence (5'–3')
hTMEM87A-WT-GFP-strep-Foward	TTC TCT CCA CAG AAG CTT ATG GCG GCG GCT GCG TGG CT
hTMEM87A-WT-GFP-strep-Reverse	GTA CAG GTT TTC CTT AAG CTC CAT TTT GGA CCT TTC AAA GTG TGT GAT CAT TCG T
hTMEM87A-WT-Forward	TCG AAT TCT GCA GTC GAC ATG GCG GCG GCT GCG TGG CT
hTMEM87A-WT-Reverse	AGG GAG AGG GGC GGA TCC CTC CAT TTT GGA CCT TTC AAA GTG TGT GAT CAT TCG T
hTMEM87A-isoform1-(GGSX3)-EGFP-Forward	AGC TCA AGC TTC GAA TTC GCC ACC ATG GCG GCG GCT GCG TGG CTT
hTMEM87A-isoform1-(GGSX3)-EGFP-Reverse	ACC ATG GTG GCG ACC GGT GAT CCG CCG CTT CCG CCG CTT CCG CCC TCC ATT TTG GAC CTT TCA AA
hTMEM87A-isoform3-(GGSX3)-EGFP-Forward	AGC TCA AGC TTC GAA TTC GCC ACC ATG GCA CAT TCC GAT ACC GT
hTMEM87A-isoform3-(GGSX3)-EGFP-Reverse	ACC ATG GTG GCG ACC GGT GAT CCG CCG CTT CCG CCG CTT CCG CCC TCC ATT TTG GAC CTT TCA AA
mTMEM87A-WT-Forward	TCG AAT TCT GCA GTC GAC ATG GCT GTG AAG ATG GCG GT
mTMEM87A-WT-Reverse	AGG GAG AGG GGC GGA TCC TTA CTC CAT CTT TGA CCT TT
hTMEM87A-isoform1 Δss-(GGSX3)-EGFP-Forward	TCA CCA CTG TCG TTT TTC AGT GCG
hTMEM87A-isoform1 Δss-(GGSX3)-EGFP-Reverse	CAT GGT GGC GAA TTC GGA GCT
shRNA-insensitive hTMEM87A WT-Forward	TAT ATT CTT GGG AAT GCT TGA GAA AG
shRNA-insensitive hTMEM87A WT-Reverse	ACG GCG CCG ATC CAA AAC TGA ATT CTC
hTMEM87A-AAA-Forward (GYG318~320AAA)	AGC AAT CGT CAA GCC ACG CCT T
hTMEM87A-AAA-Reverse (GYG318~320AAA)	GCT GCC AGA CTG ACT ATG ATG A
hTMEM87A-Y237A-Forward	GTG ATG TGT ATT GTA GCT GTC CTG TTT GGT GTT CT
hTMEM87A-Y237A-Reverse	AGA ACA CCA AAC AGG ACA GCT ACA ATA CAC ATC AC

hTMEM87A-E272A-Forward	CTG GGA ATG CTT GAG AAA GCT GTC TTC
hTMEM87A-E272A-Reverse	GAA GAC AGC TTT CGC AAG CAT TCC CAG
hTMEM87A-K273A-Forward	GGA ATG CTT GAG AAA GCT GTC TTC TAT
hTMEM87A-K273A-Reverse	ATA GAA GAC AGC TGC CTC AAG CAT TCC
hTMEM87A-E279A-Forward	GTC TTC TAT GCG GCA TTT CAG AAT ATC CGA TAC
hTMEM87A-E279A-Reverse	GTA TCG GAT ATT CTG AAA TGC CGC ATA GAA GAC
hTMEM87A-E298A-Forward	TTG ATC CTT GCA GCG CTG CTT TCA GCA
hTMEM87A-E298A-Reverse	TGC TGA AAG CAG CGC TGC AAG GAT CAA
hTMEM87A-S301A-Forward	GCA GAG CTG CTT GCA GCA GTG AAA CGC
hTMEM87A-S301A-Reverse	GCG TTT CAC TGC TGC AAG CAG CTC TGC
hTMEM87A-K304A-Forward	CTT TCA GCA GTG AAA CGC TCA CTG GCT
hTMEM87A-K304A-Reverse	AGC CAG TGA GCG TGC CAC TGC TGA AAG
hTMEM87A-R305A-Forward	TCA GCA GTG AAA GCC TCA CTG GCT CGA
hTMEM87A-R305A-Reverse	TCG AGC CAG TGA GGC TTT CAC TGC TGA
hTMEM87A-R309A-Forward	CGC TCA CTG GCT GCA ACC CTG GTC ATC
hTMEM87A-R309A-Reverse	GAT GAC CAG GGT TGC AGC CAG TGA GCG
hTMEM87A-A308M-Forward	AAA CGC TCA CTG ATG CGA ACC CTG GTC
hTMEM87A-A308M-Reverse	GAC CAG GGT TCG CAT CAG TGA GCG TTT
hTMEM87A-R257A-Forward	AGA GAT CTC CTG GCA ATT CAG TTT TGG
hTMEM87A-R257A-Reverse	CCA AAA CTG AAT TGC CAG GAG ATC TCT
hTMEM87A-R325A-Forward	ATC GTC AAG CCA GCC CTT GGA GTC ACT
hTMEM87A-R325A-Reverse	AGT GAC TCC AAG GGC TGG CTT GAC GAT
hTMEM87A-E288R-Forward	CGA TAC AAA GGA CGA TCT GTC CAG GGT
hTMEM87A-E288R-Reverse	ACC CTG GAC AGA TCG TCC TTT GTA TCG
hTMEM87A-D442A-Forward	CTG TGG GTA GAC GAT GCC ATC TGG CGC
hTMEM87A-D442A-Reverse	GCG CCA GAT GGC AGC GTC TAC CCA CAG
