# Supplementary material for: GolpHCat (TMEM87A), a unique voltage-dependent cation channel in Golgi apparatus, contributes to Golgi-pH maintenance and hippocampus-dependent memory
Source: Nat Commun. 2024 Jul 11;15:5830. doi: 10.1038/s41467-024-49297-8 (PMC11239671; doi:10.1038/s41467-024-49297-8)
Supplement: Supplementary file 3 — Description of Additional Supplementary Files [file 41467_2024_49297_MOESM3_ESM.pdf]

Description of Additional Supplementary Files

GolpHCat (TMEM87A), a unique voltage-dependent cation channel in Golgi apparatus, contributes to Golgi-pH maintenance and hippocampus-dependent memory

Hyunji Kang,[1,2,7] Ah-reum Han,[3,7] Aihua Zhang,[4] Heejin Jeong,[5] Wuhyun Koh,[1] Jung Moo Lee,[1] Hayeon Lee,[1] Hee Young Jo,[5] Miguel A. Maria-Solano,[4] Mridula Bhalla,[1] Jea Kwon,[1] Woo Suk Roh,[1] Hyun Jun Jang,[1] Jimin Yang,[3] Boyoung Lee,[1] Sun Choi,[4] Hyun Joo An,[5] Ho Min Kim,[3,6,*] C. Justin Lee[1,2,8,*]

[7] These authors contributed equally: Hyunji Kang, Ah-reum Han.

*Correspondence: cjl@ibs.re.kr (C.J.L.), hm_kim@kaist.ac.kr (H.M.K.)

File Name: Supplementary Data 1

Description: Initial structures of the K^+ conformational dynamics of hTMEM87A obtained from GaMD simulations (frame 1491).

File Name: Supplementary Data 2

Description: Final structures of the K^+ conformational dynamics of hTMEM87A obtained from GaMD simulations (frame 1846).

File Name: Supplementary Data 3

Description: Initial structure of PE binding simulations of hTMEM87A (S1).

File Name: Supplementary Data 4

Description: Final structure of PE binding simulations of hTMEM87A (S7).
