# Supplementary material for: GolpHCat (TMEM87A), a unique voltage-dependent cation channel in Golgi apparatus, contributes to Golgi-pH maintenance and hippocampus-dependent memory
Source: Nat Commun. 2024 Jul 11;15:5830. doi: 10.1038/s41467-024-49297-8 (PMC11239671; doi:10.1038/s41467-024-49297-8)
Supplement: Supplementary file 8 — Reporting Summary [file 41467_2024_49297_MOESM8_ESM.pdf]

nature portfolio

Corresponding author(s): C. Justin Lee and Ho Min Kim

Last updated by author(s): May 1, 2024

Reporting Summary

Nature Portfolio wishes to improve the reproducibility of the work that we publish. This form provides structure for consistency and transparency in reporting. For further information on Nature Portfolio policies, see our Editorial Policies and the Editorial Policy Checklist.

Statistics

For all statistical analyses, confirm that the following items are present in the figure legend, table legend, main text, or Methods section.

n/a	Confirmed	
☐	☒	The exact sample size (*n*) for each experimental group/condition, given as a discrete number and unit of measurement
☐	☒	A statement on whether measurements were taken from distinct samples or whether the same sample was measured repeatedly
☐	☒	The statistical test(s) used AND whether they are one- or two-sided<br>*Only common tests should be described solely by name; describe more complex techniques in the Methods section.*
☐	☒	A description of all covariates tested
☐	☒	A description of any assumptions or corrections, such as tests of normality and adjustment for multiple comparisons
☐	☒	A full description of the statistical parameters including central tendency (e.g. means) or other basic estimates (e.g. regression coefficient) AND variation (e.g. standard deviation) or associated estimates of uncertainty (e.g. confidence intervals)
☐	☒	For null hypothesis testing, the test statistic (e.g. *F*, *t*, *r*) with confidence intervals, effect sizes, degrees of freedom and *P* value noted<br>*Give P values as exact values whenever suitable.*
☒	☐	For Bayesian analysis, information on the choice of priors and Markov chain Monte Carlo settings
☒	☐	For hierarchical and complex designs, identification of the appropriate level for tests and full reporting of outcomes
☒	☐	Estimates of effect sizes (e.g. Cohen's *d*, Pearson's *r*), indicating how they were calculated

Our web collection on statistics for biologists contains articles on many of the points above.

Software and code

Policy information about availability of computer code

Data collection	We used ZEN software for Light Microscopy blue edition (Zeiss) to collect image data from immunocytochemistry and immunohistochemistry. We used pCLAMP (version 11) and Imaging WorkBench software was used for electrophysiological experiments. Behavioral tests were assayed using Ethovision XT (Nodulus). Cryo-EM data were collected automatically using Thermo Scientific EPU v2.9 at Institute for Basic Science. Cryo-EM Data collection statistics are shown in Supplementary Table 2.
Data analysis	We used GraphPad Prism 9 (GraphPad Software, Inc) for statistical analysis. ZEN software for Light Microscopy blue edition (Zeiss) and ImageJ were used for image analysis. The Cryo-EM data collection strategy and the analysis are listed in the Methods section of the manuscript, including the list of all software used. CryoSPARC v.3.3.2, AlphaFold v2, PHENIX 1.19.2, and COOT 0.9.6 were used for data processing, model building and structure refinement. All structure figures were generated using PyMOL 2.5.2 and ChimeraX 1.2.5. 3D structures were compared using DALI. Sequence alignment was created using Clustal Omega and Espript v3. Gaussian accelerated molecular dynamics, AMBER2021 package, CHARMM-GUI, Modeller, Particle-mesh Ewald method, OpenMM, and linear interaction energy (LIE) model were used for Molecular dynamics simulation. Partek Genomics Suite (Flow ver 10.0.21.0328; copyright 2009, Partek, St Louis, MO, USA) was used for analysis of fastq files obtained from Next Generation RNA Sequencing of Human Astrocyte culture.

For manuscripts utilizing custom algorithms or software that are central to the research but not yet described in published literature, software must be made available to editors and reviewers. We strongly encourage code deposition in a community repository (e.g. GitHub). See the Nature Portfolio guidelines for submitting code & software for further information.

Data

Policy information about availability of data

All manuscripts must include a data availability statement. This statement should provide the following information, where applicable:

- Accession codes, unique identifiers, or web links for publicly available datasets
- A description of any restrictions on data availability
- For clinical datasets or third party data, please ensure that the statement adheres to our policy

The atomic coordinates for hTMEM87A, hTMEM87A with gluconate, hTMEM87A A308M have been deposited to the Electron Microscopy Data Bank and the Protein Data Bank with the accession numbers PDB: 8HSI (EMD-34998), PDB: 8HTT (EMD-35017), and PDB: 8KB4 (EMD-37069), respectively. Raw files obtained in the Next Generation RNA Sequencing experiments are available on NCBI GEO (Accession number GSE228084). Source data is provided this paper. All accessions will be freely available upon publication. All other relevant data are available from the corresponding authors.

Research involving human participants, their data, or biological material

Policy information about studies with human participants or human data. See also policy information about sex, gender (identity/presentation), and sexual orientation and race, ethnicity and racism.

Reporting on sex and gender	*Use the terms sex (biological attribute) and gender (shaped by social and cultural circumstances) carefully in order to avoid confusing both terms. Indicate if findings apply to only one sex or gender; describe whether sex and gender were considered in study design; whether sex and/or gender was determined based on self-reporting or assigned and methods used.<br>Provide in the source data disaggregated sex and gender data, where this information has been collected, and if consent has been obtained for sharing of individual-level data; provide overall numbers in this Reporting Summary. Please state if this information has not been collected.<br>Report sex- and gender-based analyses where performed, justify reasons for lack of sex- and gender-based analysis.*
Reporting on race, ethnicity, or other socially relevant groupings	*Please specify the socially constructed or socially relevant categorization variable(s) used in your manuscript and explain why they were used. Please note that such variables should not be used as proxies for other socially constructed/relevant variables (for example, race or ethnicity should not be used as a proxy for socioeconomic status).<br>Provide clear definitions of the relevant terms used, how they were provided (by the participants/respondents, the researchers, or third parties), and the method(s) used to classify people into the different categories (e.g. self-report, census or administrative data, social media data, etc.)<br>Please provide details about how you controlled for confounding variables in your analyses.*
Population characteristics	*Describe the covariate-relevant population characteristics of the human research participants (e.g. age, genotypic information, past and current diagnosis and treatment categories). If you filled out the behavioural & social sciences study design questions and have nothing to add here, write "See above."*
Recruitment	*Describe how participants were recruited. Outline any potential self-selection bias or other biases that may be present and how these are likely to impact results.*
Ethics oversight	*Identify the organization(s) that approved the study protocol.*

Note that full information on the approval of the study protocol must also be provided in the manuscript.

Field-specific reporting

Please select the one below that is the best fit for your research. If you are not sure, read the appropriate sections before making your selection.

☒ Life sciences ☐ Behavioural & social sciences ☐ Ecological, evolutionary & environmental sciences

For a reference copy of the document with all sections, see nature.com/documents/nr-reporting-summary-flat.pdf

Life sciences study design

All studies must disclose on these points even when the disclosure is negative.

Sample size	No statistical method was used to predetermine sample size. Sample sizes were determined based on previous experiences or a review of similar experiments in the literature.<br>For Cryo-EM, A total of 10,377 movies for hTMEM87A and 13,099 movies for hTMEM87A-Gluc were acquired using the EPU software. The data were sufficient to yield high-resolution cryo-EM maps density. The final structures were calculated with a set of 445,198 for hTMEM87A and 201,915 for hTMEM87A-Gluc particle images, respectively. The detailed of cryo-EM data sets are given in Methods and Supplementary information.
Data exclusions	Grubb's test were used to exclude significant outliers. Additionally, data was excluded from behavior test due to the technical issue, such as unsuccessful surgery determined by virus expression in non-targeted area or mis-localization of cannula. In figure 8n-8q, two mice in scrambled group were excluded due to virus expression in non-targeted area and mis-localization of cannula.

Replication	All experiments were done at least three biological replicates (cell and proteoliposome preparation for electrophysiological experiments, multiple brain sample preparation for electrophysiological experiments and multi-omics, and mice for behavior test).
Randomization	For Cryo-EM, particle images were automatically selected from averaged micrographs. 2D templates and the initial model were derived from within the data sets. The resolution of the final structure was calculated according to the gold-standard FSC. For behavior test after virus injection, mice were randomly assigned to the groups (Scrambled or GolpHCat shRNA).
Blinding	All Cryo-EM data acquisition and processing steps were performed in an unbiased and automated manner according to the current standards in the field of cryo-EM. To perform behavioral experiments in a blinded manner, experiments and data analysis were performed by different authors (H.K. and H.L.).

Reporting for specific materials, systems and methods

We require information from authors about some types of materials, experimental systems and methods used in many studies. Here, indicate whether each material, system or method listed is relevant to your study. If you are not sure if a list item applies to your research, read the appropriate section before selecting a response.

Materials & experimental systems

n/a	Involved in the study
☐	☒ Antibodies
☐	☒ Eukaryotic cell lines
☒	☐ Palaeontology and archaeology
☐	☒ Animals and other organisms
☒	☐ Clinical data
☒	☐ Dual use research of concern
☒	☐ Plants

Methods

n/a	Involved in the study
☒	☐ ChIP-seq
☒	☐ Flow cytometry
☒	☐ MRI-based neuroimaging

Antibodies

Antibodies used	For immunocytochemistry and histochemistry, primary and secondary antibodies used are as follow: rabbit anti-TMEM87A (1:100, Novus Biologicals, NBP1-90531), mouse anti-Giantin (1:100, abcam, ab37266), mouse anti-Golgin-97 (1:100, invitrogen, A-21270), chicken anti-GFAP (1:200, Millipore, AB5541), guineapig anti-NeuN (1:200, Millipore, ABN90), anti-rabbit IgG Alexa Fluor 488 (1:500, Jackson lab, 711-547-003), anti-mouse IgG Alexa Fluor 594 (1:500, Jackson lab, 715-585-150), anti-chicken IgG Alexa Fluor 594 (1:500, Jackson lab, 703-585-155), anti-chicken IgG Alexa Fluor 647 (1:500, Jackson lab, 703-605-155), and anti-guineapig IgG Alexa Fluor 647 (1:500, Jackson lab, 706-605-148).<br>For western blotting, primary antibodies used are as follow: rabbit anti-TMEM87A (1:1000, Novus Biologicals, NBP1-90531), rabbit anti-β-actin (1:2000, Abcam, ab133626), and mouse anti-GAPDH (1:1000, Abcam, ab8245), antirabbit HRP (Pierce, 170-6515), and anti-mouse HRP (Pierce, 170-6516).
Validation	Anti-TMEM87A was validated in this paper using KO mice. Anti-Giantin was validated in previous reports including Petrosyan A, et al (Mol Cancer Res 12:1704-16, 2014). Anti-Giantin was validated in previous reports including Amagai, Yuta, et al (Nature communications 14.1 (2023): 2683. Anti-GFAP and anti-NeuN were validated previous reports including (Jo, et al., Nat. Med. 20, 886–896, 2014). Anti-β-actin was validated previous reports including (Han, et al., Experimental neurobiology, 28.2:183, 2019). Anti-GAPDH was validated previous reports including Kim, Tai Young, et al (Antiviral Research 208 (2022): 105428).

Eukaryotic cell lines

Policy information about cell lines and Sex and Gender in Research

Cell line source(s)	Chinese hamster ovary-K1 (CHO-K1, referred to as CHO) cells and immortalized human fetal astrocytes (SV40) were purchased from the Korean Cell Line Bank and abm (T0280, Richmond, BC, Canada), respectively. For protein expression, Expi293F cells (Thermo Fisher, Cat#A14527) were used.
Authentication	None of the cell lines used have been authenticated.
Mycoplasma contamination	All cell lines tested negative for mycoplasma contamination
Commonly misidentified lines<br>(See ICLAC register)	none.

Animals and other research organisms

Policy information about studies involving animals; ARRIVE guidelines recommended for reporting animal research, and Sex and Gender in Research

Laboratory animals	We used 6~7 weeks old of C57BL/6J mice and 9~15 weeks old GolpHCat KO (GHBio) and WT littermates in C57BL/6J background were used.
Wild animals	This study did not involve wild animals.
Reporting on sex	We used male mice in this study.
Field-collected samples	This study did not involve samples collected from the field.
Ethics oversight	Animal use and procedures were approved by Institutional Animal Care and Use Committees (IACUCs) of IBS (Daejeon, Korea).

Note that full information on the approval of the study protocol must also be provided in the manuscript.

Plants

Seed stocks	*Report on the source of all seed stocks or other plant material used. If applicable, state the seed stock centre and catalogue number. If plant specimens were collected from the field, describe the collection location, date and sampling procedures.*
Novel plant genotypes	*Describe the methods by which all novel plant genotypes were produced. This includes those generated by transgenic approaches, gene editing, chemical/radiation-based mutagenesis and hybridization. For transgenic lines, describe the transformation method, the number of independent lines analyzed and the generation upon which experiments were performed. For gene-edited lines, describe the editor used, the endogenous sequence targeted for editing, the targeting guide RNA sequence (if applicable) and how the editor was applied.*
Authentication	*Describe any authentication procedures for each seed stock used or novel genotype generated. Describe any experiments used to assess the effect of a mutation and, where applicable, how potential secondary effects (e.g. second site T-DNA insertions, mosiacism, off-target gene editing) were examined.*
